# Importance of Circ0009910 in colorectal cancer pathogenesis as a possible regulator of miR-145 and PEAK1

**DOI:** 10.1186/s12957-021-02378-0

**Published:** 2021-09-03

**Authors:** Sepideh Kadkhoda, Reza Taslimi, Farshid Noorbakhsh, Farzaneh Darbeheshti, Javad Tavakkoly Bazzaz, Soudeh Ghafouri-Fard, Abbas Shakoori

**Affiliations:** 1 Department of Medical Genetics, School of Medicine, Tehran University of Medical Sciences, Tehran, Iran; 2 Department of Gastroenterology, Imam Khomeini Hospital, Tehran University of Medical Sciences, Tehran, Iran; 3 Department of Immunology, School of Medicine, Tehran University of Medical Sciences, Tehran, Iran; 4 Medical Genetics Network (MeGeNe), Universal Scientific Education and Research Network (USERN), Tehran, Iran; 5 Department of Medical Genetics, Shahid Beheshti University of Medical Sciences, Tehran, Iran; 6 Department of Medical Genetics, Cancer Institute of Iran, Imam Khomeini Hospital Complex, Tehran University of Medical Sciences, Dr. Qarib St., Keshavarz Blvd, Tehran, Iran

**Keywords:** Colorectal cancer, Circ0009910, miR-145-5p, PEAK1, Non-coding RNA

## Abstract

**Introduction:**

Colorectal cancer (CRC) is one of the most frequent neoplasms in the world. Based on the emerging role of noncoding RNAs, particularly circular RNAs in pathogenesis of cancers, we designed this study to inspect the expression levels of a circ0009910-mediated regulatory pathway in colorectal cancer.

**Methods:**

After bioinformatics analyses and construction of putative circ0009910/ miR-145-5p/PEAK1 pathway, the expression levels of these components were evaluated in 50 CRC tissues and adjacent specimens by quantitative real-time PCR. Moreover, we appraised the correlation coefficients between these transcripts and calculated the correlation between circ0009910 expression levels with clinicopathological features of patients.

**Results:**

Circ0009910 and PEAK1 were significantly upregulated, while miR-145-5p was decreased in CRC samples compared with adjacent tissues (*p* < 0.05). Moreover, statistically significant correlations were observed between expression levels of circ0009910, miR-145-5p, and PEAK1. We also reported considerable correlations between circ0009910 expression and clinicopathological parameters including sex and perineural invasion. Finally, ROC curve analysis showed circ0009910 level as a discriminative biomarker for CRC.

**Conclusion:**

For the first time, we could introduce circ0009910 as an important biomarker in CRC. Collectively, this investigation helped us to identify a newly diagnosed pathway in CRC that can be a potential axis for designing effective drugs for treatment of CRC patients.

**Supplementary Information:**

The online version contains supplementary material available at 10.1186/s12957-021-02378-0.

## Introduction

Among all malignancies, colorectal cancer (CRC) is the third most frequent neoplasm and it ranks fourth in terms of cancer deaths [1]. Australia and New Zealand have the highest rates of CRC [2]. Unfortunately, the prevalence of this disease has increased in most parts of the world with the change of people’s lifestyle, particularly inappropriate eating habits, obesity, adopting a machine life, and increasing consumption of tobacco and alcohol [3]. A previous systematic review and meta-analysis has shown that incidence rate of CRC has raised in Iran possibly as a result of increasing personal and environmental risk factors in addition to enhancement of cancer registries and availability of health services [4]. Age-standardized incidence values in Iran have been reported to be 8.16 and 6.17 for men and women, respectively [4]. CRC often occurs in a sporadic form, but a percentage of cases are inherited or due to inflammatory bowel disease [5]. This malignancy is heterogeneous and genetic and environmental factors partake in this disease [6]. Despite recent advances in the diagnosis and treatment, the prognosis of CRC is still poor because of late detection, metastasis to distant organs, and disease recurrence. From a genetic standpoint, the discovery of potentially effective biomarkers contributing in CRC pathogenesis can contribute in faster detection of at-risk individuals and increasing of their life expectancy [7]. A recent study has shown the importance of long non-coding RNAs in the pathogenesis of CRC and introduced a prognosis model for CRC based on expression profile of six of these transcripts [8]. Another study has designed a prognostic predictor for CRC through comprehensive assessment of alternatively spliced transcripts [9]. Other bioinformatics methods have identified a 12-gene signature that can predict prognosis of CRC with high efficiency [10]. Moreover, combined assessment of expression of RNA-binding proteins in CRC samples has led to identification prediction models for this malignancy [11]. Among genes which are associated with CRC prognosis are Na+/Ca2+ exchangers [12], ferroptosis-related genes [13], and those participating in chemokine signaling and cytokine–cytokine receptor interactions [14].

Circular RNAs as emerging group of stable non-coding RNAs with circular configuration play significant roles in the pathogenesis of various disorders, particularly cancers [15]. So, they have attracted the attention of many researchers in the field of biomarker discovery. Yet, research in this area is still in its infancy [16]. They are formed through construction of a closed loop between two ends of their host genes. They can act as molecular sponges for miRNAs [17]. Although they were initially thought as error products of splicing, their importance in diverse cellular processes was gradually realized [18]. For instance, circ_0055625 has been found to participate in the pathogenesis of CRC and response to radiotherapy through influencing miR-338-3p/MSI1 axis [19]. miRNAs as another non-coding RNAs play important roles in genes expression modification. These small RNAs act as oncogene or tumor suppressors and regulate expression of genes through binding to 3′ untranslated regions of target genes [20]. It is estimated that expression of 60% of genes is influenced by miRNAs [21, 22]. Based on the bioinformatics analysis from NCBI Gene Expression Omnibus (GEO) and different databases, we predicted the presence of regulatory axis, namely, circ0009910/miR-145-5p/PEAK1 axis. The length of exonic circ0009910 is 315 base pairs that are produced from *MFN2* located on chromosome 1 as the host gene. *PEAK1* (also known as *KIAA2002*) is located on 15q24.3. The protein has a molecular mass of 193106 Da, is a member of NFK3 family, and acts as a tyrosine kinase. This kinase is functionally associated with actin protein and thereupon, has a role in cell connections and migrations [23].

In the recent studies, the role of circ0009910 as an oncogene has been discovered in a few cancers [24]. Yet, it has not been studied in CRC. So, we designed this study to quantify the expression level of circ0009910, miR-145-5p, and PEAK1 in CRC tumors compared to adjacent tissues. Also, the relationship between expression of these genes and the clinical parameters of the patients such as age, gender, tumor diameter, stage, grade, and other features was explored.

## Methods

### Bioinformatics analysis

In a recent study, we have analyzed GPL19978 platform from GEO microarray dataset (GSE126095) and detected 37 differentially expressed circRNAs between cancer and non-cancer tissues using a cut-off point of adjusted *P* value < 0.05 and log fold change ≥|3| (supplementary table 1) [25].

In the current study, the exonic circ0009910 (circ100053) as a differentially expressed (DE) circular RNA between colorectal tumors and adjacent tissues was selected (log Fc = 3.03281 and adj *p* value = 2.06E−05). Then, GSE128449 (platform GPL14767) was analyzed to determine DEmiRNAs between colorectal tumors and adjacent tissues. In this step, DEmiRNAs with adjusted *P* value < 0.05 and log fold change ≥|3| were picked out.

Using Circular RNA Interactome database, it was found that circ0009910 has connection site for 17 miRNAs (supplementary table 2). Through investigating this database and GSE128449, miR-145-5p as a DEmiRNA and target of this circular RNA was selected (log Fc = −4.14554 and adj *p* value = 4.10E−05). For the selection of DEmRNAs between colorectal tumors and adjacent tissues, GSE41657 (platform GPL6480) was analyzed and DEmRNAs that had the criteria of log fold change ≥|2| and adjusted *P* value < 0.05 were considered.

*PEAK1* gene (pseudopodium enriched atypical kinase 1) was diagnosed as a target of miR-145-5p through analysis of GSE41657 and assessment of TargetScan and miRmap databases (log Fc = 2.313187 and adj *p* value= 5.12E−05). Then, circRNA/miRNA/mRNA pathway was organized for subsequent studies. We hypothesized that circ0009910 regulates PEAK1 through sponging miR-145-5p.

**Fig. 1:**
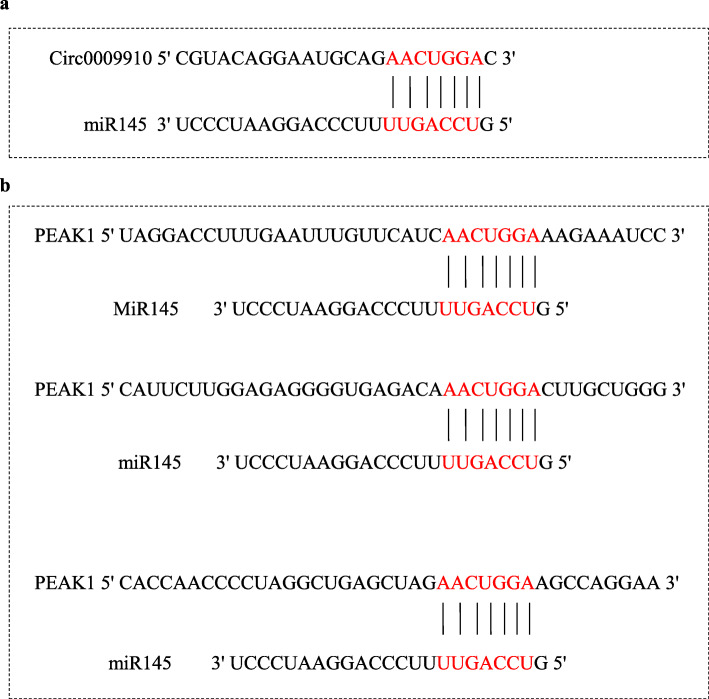
**a** One interaction site between circ0009910 and miR-145-5p according to Circular RNA Interactome database. **b** Three interaction sites between miR-145-5p and PEAK1 according to miRmap database

The interaction site between circ0009910 and miR-145-5p and also three interaction sites between miR-145-5p and PEAK1 are illustrated in Figs. 1 a and b, respectively.

### Tissue samples collection

First, fifty paired specimen of CRC tissues and adjacent corresponding tissues were purchased from tumor bank of Imam Khomeini Hospital (Tehran, Iran). The Ethical Committee of Tehran University of Medical Sciences approved this study (Ethical code: IR.TUMS.MEDICINE.REC.1399.590). These samples were taken from people of Iranian descent and none of them received chemotherapy and radiotherapy before surgery. Moreover, 10 normal colorectal tissues were obtained. The written consent form was signed by these individuals. The clinicopathological information such as age, gender, tumor size, stage and grade of tumors, invasion status, clinical metastasis, smoking history, and family history of CRC were also obtained. All tissues were kept at −70 °C.

### RNA extraction and cDNA synthesis

The Riboex (geneall, Korea) was used to extract total RNA from tumors, adjacent tissues, and normal tissues following the instructions of the solution. The quantity of extracted RNAs was assessed by the Nanodrop 2000C (Thermo Scientific, USA). RNA was also subjected to electrophoresis on 1.5% agarose gel for RNA quality assessment. Then, cDNA was produced from 50 μg of RNAs by cDNA synthesis kit (Yekta Tajhiz Azma, Iran). The specific stem loop RT primer for miR-145-5p and snRNA U6 was added to the reaction mixture. The resulting mixture for production of cDNA was subjected to the following temperature conditions: 15 min at 37 °C, 60 min at 42 °C for, and then 5 min at 70 °C.

### Quantitative real-time PCR

The suitable primers designed with help of Circular RNA Interactome, Primer_Blast and Gene Runner software. Moreover, the circPrimer 1.2 software was used to assess the specificity of circRNA primers. The sequences of primers are displayed in Table 1.

**Table 1:** Sequences of primers

Primer name	Primer type	Sequence (5′→ 3′)
**circ0009910**	Forward	TTTGGCCGCGCAATGTCC
	Divergent reverse	GCATTCACCTCAGCCATGTGTC
**miR-145-5p**	Stem loop RT	GTCGTATCCAGTGCAGGGTCCGAGGTATTCGCACTGGATACGACAGGGAT
	Forward	GGCTTAGTCCAGTTTTCCCAG
	Universal reverse	GTGCAGGGTCCGAGGT
**PEAK1**	Forward	CTATGGACCCGAACCCTTGTAG
	Reverse	GGTTGGGAAGCATTGGGTG
**SnRNA U6**	Stem loop RT	AACGCTTCACGAATTTGCGT
	Forward	CTCGCTTCGGCAGCACA
	Reverse	AACGCTTCACGAATTTGCGT
**B2M**	Forward	CCACTGAAAAAGATGAGTATGCCT
	Reverse	CCAATCCAAATGCGGCATCTTCA

Relative expressions of circRNA0009910, miR-145-5p, and PEAK1 were measured in all tissues using RealQ Plus 2× Master Mix Green low Rox (Ampliqon, Denmark). Reactions were performed in the LightCycler 96 Real-Time PCR System (Roche, Germany). All of the qPCR reactions were performed in duplicate. *B2M* was used as the reference gene for circRNA0009910 and *PEAK1*. snRNA U6 was selected as the reference gene for miR-145-5p. Thermal cycling conditions for miR-145-5P and *PEAK1* consisted of preincubation at 95 °C for 900 s followed by amplification in 40 cycles at 95 °C for 15 s and 60 °C for 45 s. The suitable annealing temperature for circ0009910 was 59 °C. The PCR products were run on 2% agarose gel. The back splice junction of circ0009910 was verified by Sanger sequencing. For determination of the fold change of the genes, 2^−ΔΔCT^ formula was applied. In this study, normal colorectal tissues were used as calibrator for the relative expression analysis.

### Statistical analysis

All statistical analysis in this study was accomplished by the GraphPad Prism 8.0 (GraphPad Software, Inc., San Diego, CA) software. The relative expression of circ0009910, miR-145-5p, and PEAK1 was evaluated in colorectal tumors and adjacent tissues using the paired sample *t* test. The correlation of expression of these three genes was measured using Spearman correlation coefficient. Association between expression of circ0009910 and clinicopathological features of patients was calculated by Mann-Whitney and one-way ANOVA tests (Kruskal-Wallis). Also, the receiver operating characteristic (ROC) curve was illustrated by the GraphPad Prism v.8 software. The *p* value < 0.05 was considered to define the statistical significance in all of measurements.

## Results

### Expression analysis of circ0009910, miR-145-5p, and PEAK1

**Fig. 2:**
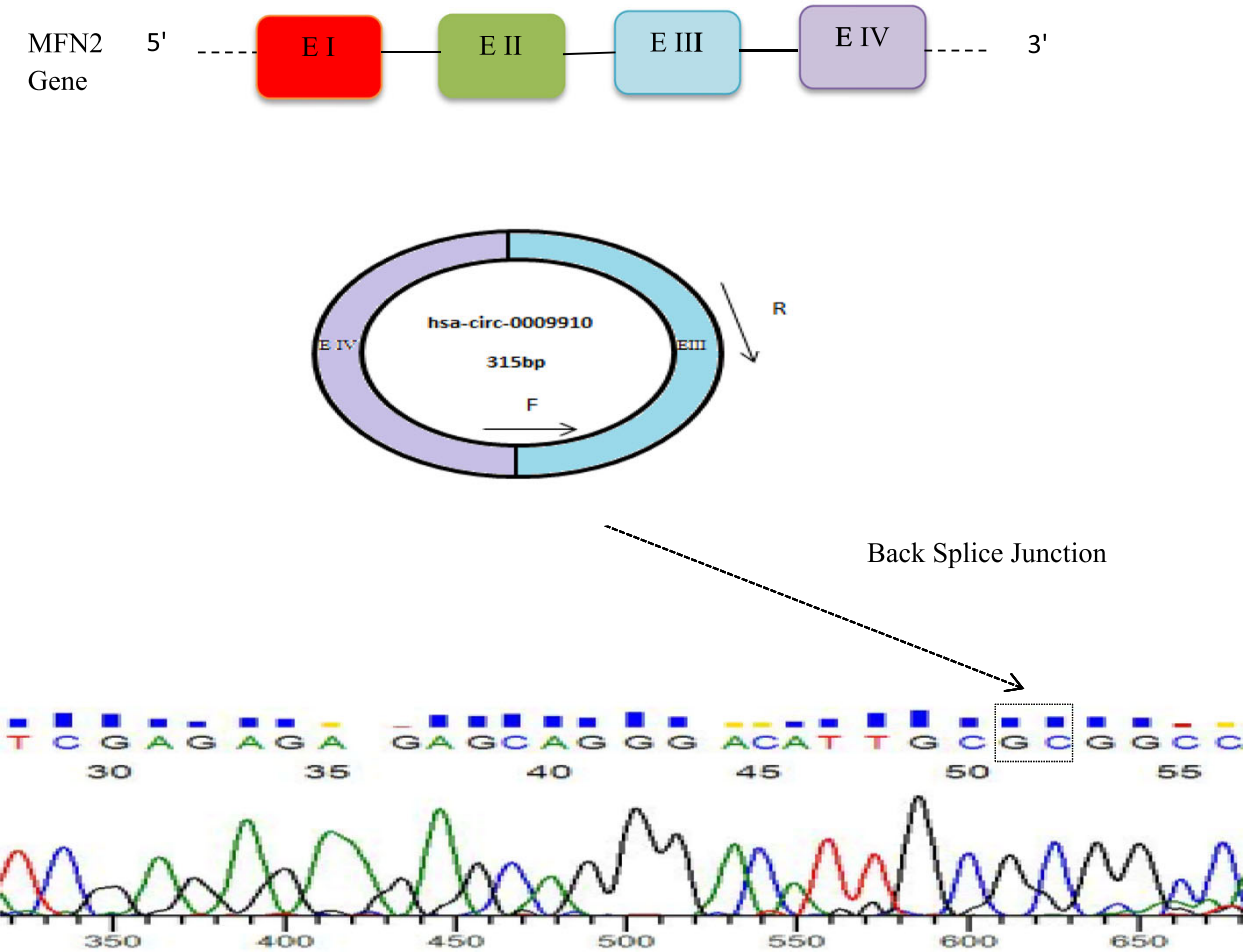
The schematic image of circ0009910 production from MFN2 gene on chromosome 1. The back-splice intersection location of circ0009910 was verified by Sanger sequencing

By the Sanger sequencing back splice junction arising from connecting of exon 3 and exon 4 of *MFN2* gene as host gene of circ0009910 was verified (Fig. 2).

**Fig. 3:**
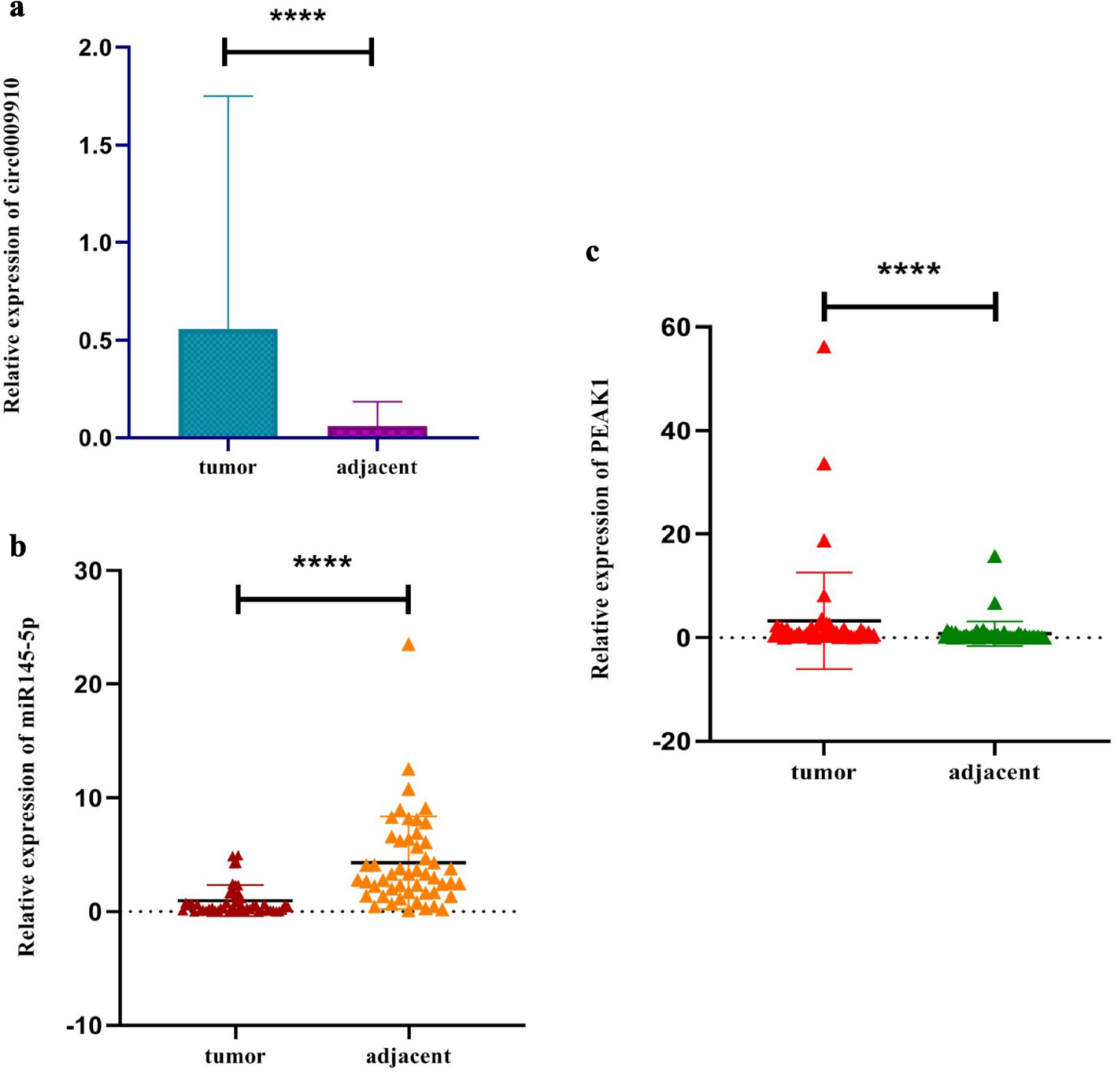
The relative expression of circ0009910 (**a**), miR-145-5p (**b**), and PEAK1 (**c**) in colorectal cancer relative to adjacent tissues. Asterisks indicate significant difference between two mentioned groups (*P* value = < 0.0001)

We found considerable upregulation of circ0009910 in colorectal tumors compared with adjacent tissues (*P* value < 0.0001) (Fig. 3a). This finding supported GSE126095 results. Moreover, significant decrease of miR-145-5p (*P* value < 0.0001) and increase of PEAK1 (*P* value < 0.0001) levels were observed in colorectal tumors compared with adjacent tissues (Fig. 3b and c).

### Correlation analysis between expression levels of circ0009910, miR-145-5p, and PEAK1

**Fig. 4:**
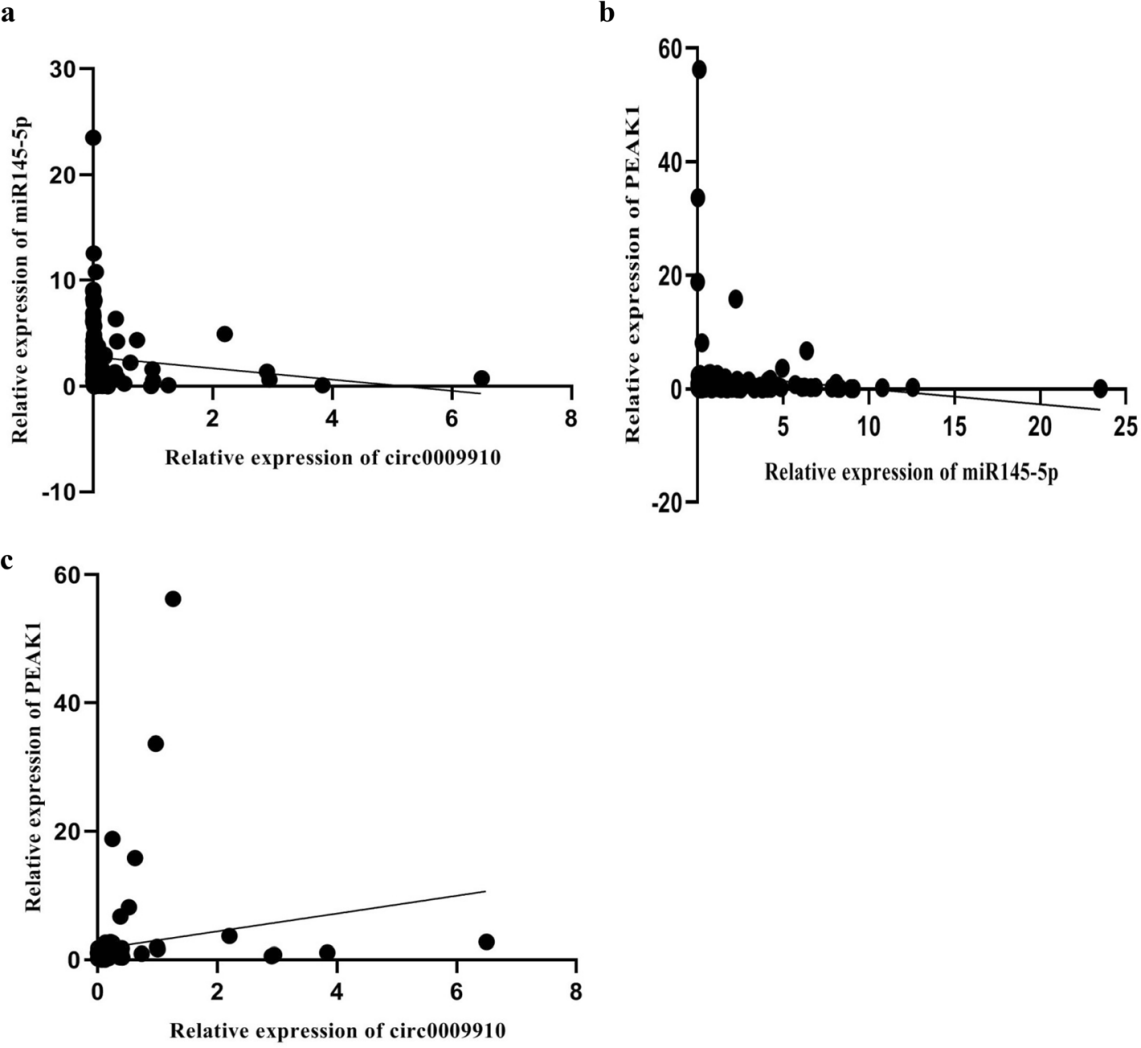
**a** The negative association between circ0009910 and miR-145-5p expression in CRC (*r* = −0.387, *P* value < 0.0001). **b** The negative association between miR-145-5p and PEAK expression in CRC (*r* = −0.213, *P* value = 0.033). **c** The positive association between circ0009910 and PEAK1 expression in CRC (*r* = 0.649, *P* value < 0.0001)

Correlation analysis between expression levels of circ0009910, miR-145-5p, and PEAK1 was accomplished by Spearman correlation coefficient. Results showed negative significant correlation of circ0009910 and miR-145-5p (*r* = −0.387 and *P* value < 0.0001) (Fig. 4a). Also, our study showed that there is a negative considerable correlation between miR-145-5p and PEAK1 (*r* = −0.213 and *P* value = 0.033) (Fig. 4b).

Moreover, positive significant correlation of circ0009910 with PEAK1 was demonstrated (*r* = 0.649 and *P* value < 0.0001) (Fig. 4c).

**Fig. 5:**
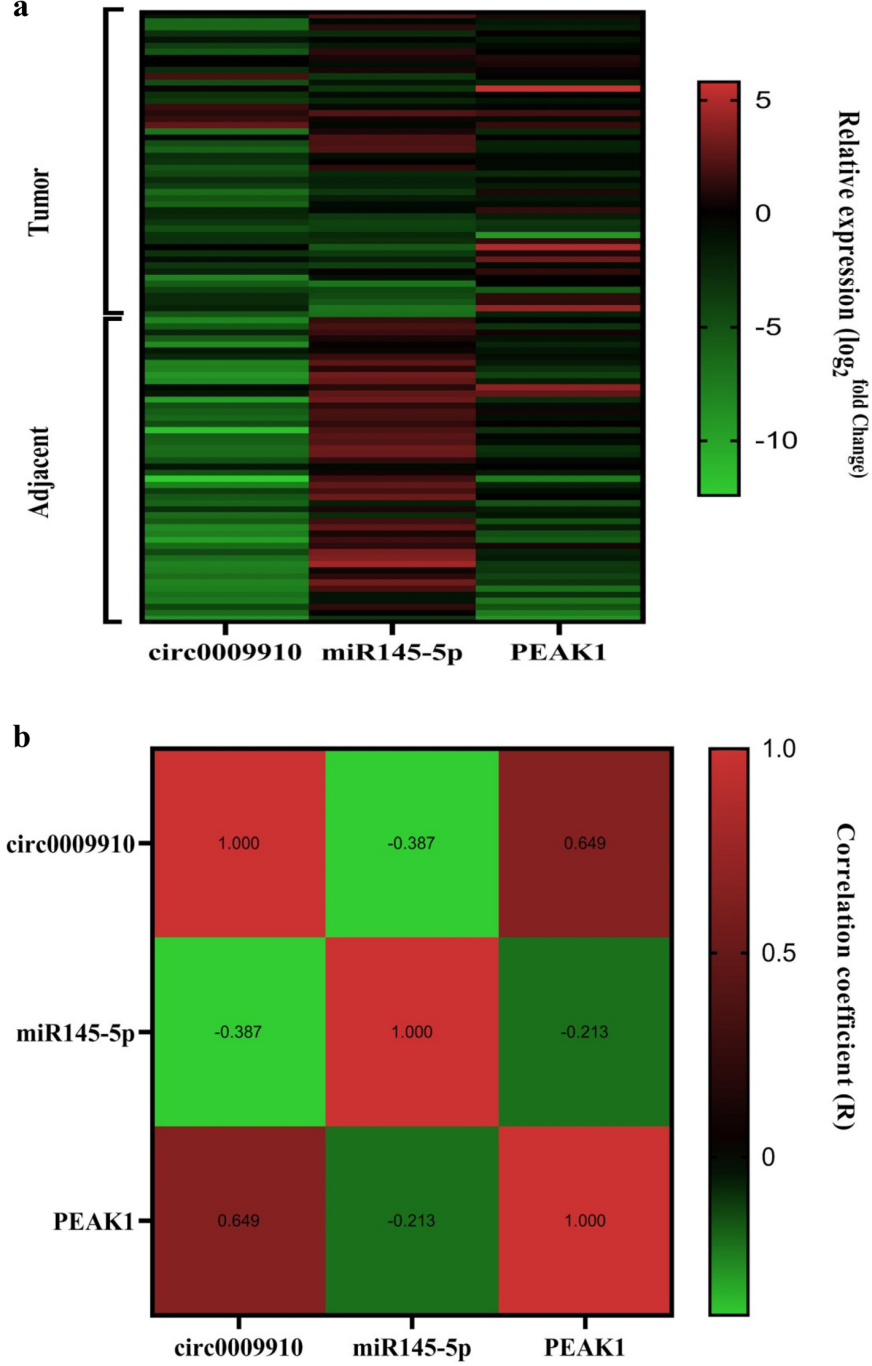
(**a**) The grouped graph of expression correlation of circ0009910 (**a**), miR-145-5p (**b**), and PEAK1 (**c**) in colorectal cancer and adjacent tissue as computed by nonparametric Spearman correlation. (**b**) Hierarchical clustering showing expression values (log2 transformed) of circ0009910, miR-145-5p, and PEAK1 in CRC samples compared with adjacent colorectal tissues. Red strips indicate high relative expression and green strips indicate low relative expression. The rows and columns show samples and transcripts respectively

Thus, according to expression correlations between these two non-coding RNA and their down-stream gene, our hypothesis was proved. Finally, we depicted hierarchical clustering showing expression values (log2 transformed) of circ0009910, miR-145-5p, and PEAK1 in CRC samples relative to non-cancerous colorectal tissues (Fig. 5).

### The importance of circ0009910 as a valuable biomarker in colorectal cancer

**Fig. 6:**
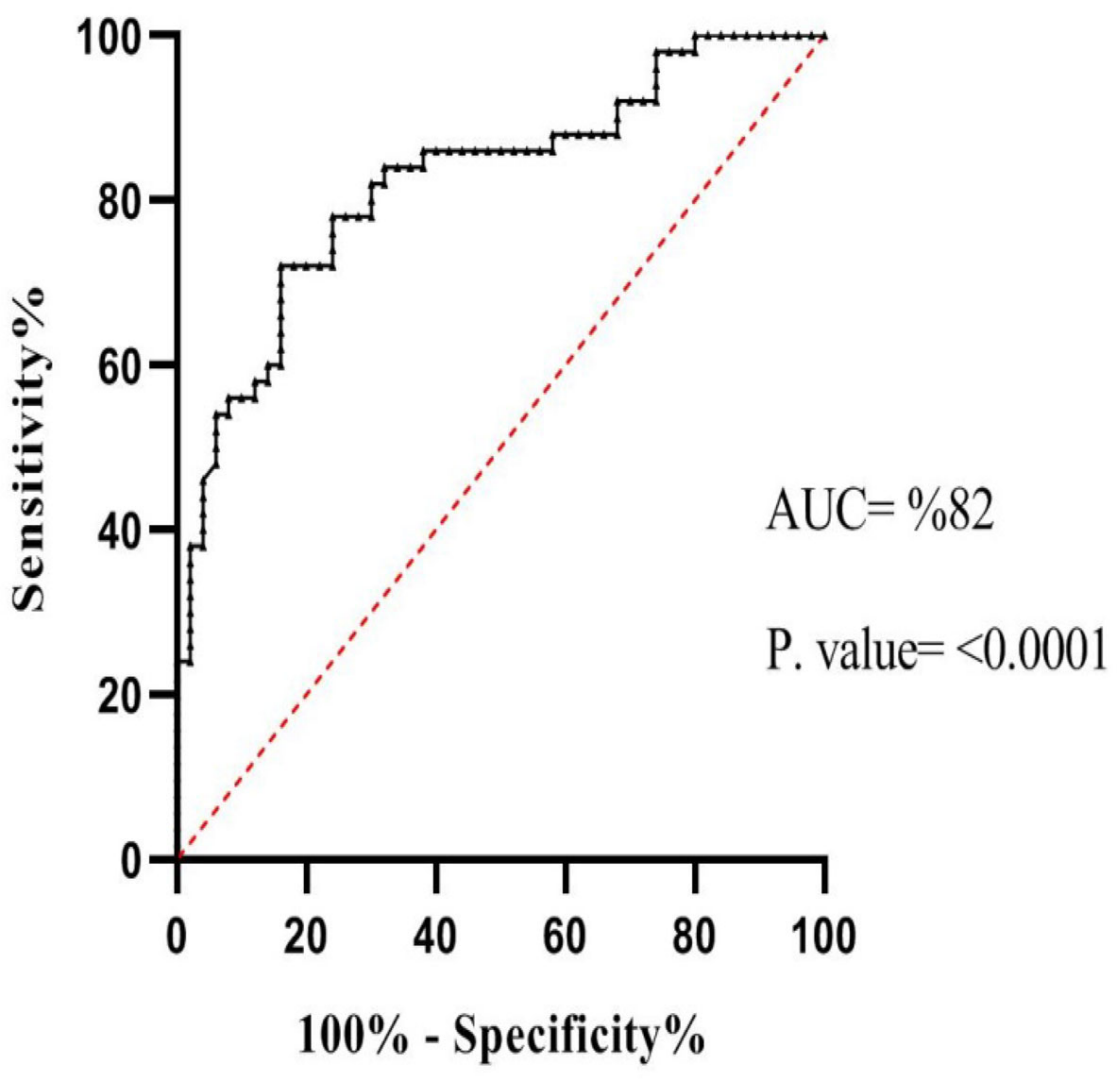
The receiver operating characteristic (ROC) curve of circ0009910 expression for discrimination of colorectal tumors from adjacent tissues. AUC indicates area under the ROC curve

We used 2^–delta Ct values (Ct Target gene-Ct Normalizer) in adjacent tissues and tumoral tissues, separately. Then, we used these values for making ROC curves. ROC curve analysis verified the possible clinical efficiency of circ0009910 in CRC as a distinctive biomarker. This evaluation revealed that circ0009910 can be proposed as a valuable biomarker for CRC (AUC = 0.82; *P* value < 0.0001) with 72% sensitivity and 84% specificity in the cutoff value < 0.025 (Fig. 6).

### Correlation analysis between expression levels of circ0009910 and clinicopathological features of CRC patients

The clinicopathological data of patients as age, gender, tumor size, grade and stage and etc., are demonstrated in Table 2. Clinicopathological analyses showed that upregulation of the circ0009910 in colorectal tumors are associated with gender (*P* value = 0.0108) and perineural invasion (*P* value = 0.0301).

**Table 2:** The association between of circ0009910 expression and clinicopathological features of CRC patients. There is significant correlation between circ0009910 level and gender of patients and perineural invasion (*P* value < 0.05)

Parameters	Subclasses	Number of patients (%)	Median of circ0009910 expression	***P*** value
**Gender**	Female	19 (38%)	0.03	**0.0108**
	Male	31 (62%)	0.14	
**Age (years)**	< 50	23 (46%)	0.14	0.0832
	≥ 50	27 (54%)	0.08	
**Tumor diameter**	< 2	1 (2%)	0.40	0.2504
	2-5	26 (52%)	0.27	
	> 5	23 (46%)	0.88	
**Grade**	I	13 (26%)	0.66	0.4557
	II	30 (60%)	0.62	
	III	7 (14%)	0.16	
**Lymphatic invasion**	Yes	39 (78%)	0.12	0.3302
	No	11 (22%)	0.04	
**Vascular invasion**	Yes	39 (78%)	0.12	0.3302
	No	11 (22%)	0.04	
**Perineural invasion**	Yes	9 (18%)	0.73	**0.0301**
	No	41 (82%)	0.11	
**Clinical stage**	I	1 (2%)	1.003	0.4094
	II	24 (48%)	0.67	
	III	17 (34%)	0.39	
	IV	8 (16%)	0.50	
**Family history**	Yes	15 (30%)	0.14	0.8342
	No	35 (70%)	0.11	
**Smoking**	Yes	8 (16%)	0.13	0.7065
	No	42 (84%)	0.11	

## Discussion

Nowadays, the role of noncoding RNAs such as miRNAs, long non-coding RNAs, and circular RNAs has been recognized in the pathogenesis of different diseases including cancer [26]. CRC is one of the most frequent neoplasms in the world and 1.9 million new CRC patients were diagnosed in 2020 [27]. So far, the involvement of a number of circular RNAs including ciRS-7-A [28], circITGA7 [29], PIP5K1A [30], GLIS2 [31], and HIPK3 [32] in the development of colorectal cancer has been identified.

The oncogenic role of circ0009910 has been studied in limited types of tumors, but no studies have been done in CRC. According to Li study in 2019, overexpression of circ0009910 in patients with ovarian cancer has been associated with poor prognosis. In this type of cancer, circ0009910 has been found to target miR-145 and act as a sponge for this miRNA [33]. In hepatocellular carcinoma, circ0009910 has been identified as an oncogenic RNA. This circRNA had negative correlation with miR-335-5p and promoted proliferation, invasion, and metastasis of these cells. As ROCK1 expression is influenced by miR-335-5p, circ0009910 can indirectly regulate expression of ROCK1 [34]. In gastric cancer, expression of circ000910 has been associated with advanced stage, undesirable overall survival and higher possibility of metastasis. On the other hand, its inhibition has reduced cell growth and aggressive power of tumor, increased expression of E-cadherin and reduced expression of snail, N-cadherin, and mesenchymal elements [24].

In patients with CML who developed resistance to imatinib, circ0009910 acts as a sponge for miR34a-5p and activates autophagy mechanism, thus reducing the life expectancy of patients [35]. In AML, upregulation of circ0009910 has been associated with weak prognosis of the patients. Moreover, this non-coding RNA sponges miR20a-5p and inhibits apoptosis [36].

Consistent with this report, Deng et al. have discovered the effects of circ0009910 on miR-449a-IL6R/BCL2/BAX pathway regulate cell proliferation, cell immortality, and resistance to cell cycle inhibitors [37]. According to Feng et al.’s investigation, miR-145 is downregulated in colorectal tumor specimens in association with poor clinical outcome. Artificial enhancement of miR-145 expression in colorectal cell lines led to decreasing of cell proliferation, motility, and invasion. In addition, this small non-coding RNA inhibited Fascin-1 [38]. In breast cancer, ZMYND10 via increasing expression of miR-145 and downregulation of NEDD9 suppressed proliferation, migration, and invasion of cells [39].

Negative expression of PEAK1 in gastric tumors and its relationship with tumor grade, invasion, and lymph node metastasis showed the role of this gene in epithelial to mesenchymal transition (EMT) [40]. However, Ding et al. have reported upregulation of PEAK1 in lung cancer and its influence on ERK1/2 and JAK2 signaling as well as expressions of MMP2, MMP9, EMT, and metastasis [41]. In colorectal tumors, two different studies have reported conflicting results about the role of PEAK1 gene. First, authors have reported downregulation of PEAK1 and significant association between its downregulation and larger tumor diameter, poor differentiation, high probability of metastasis, and advanced stage. Moreover, they demonstrated positive correlation between PEAK1 expression and PPP1R12B and its inhibitory effect on Grb2/PI3K/Akt pathway [42]. In another study, authors have reported oncogenic role for PEAK1 in CRC. Moreover, they have demonstrated the role of EGFR/KRas signaling axis and miR-181d on its expression [43].

In this survey, we examined expression of circ0009910/miR-145-5p/PEAK1 axis in fifty pairs of colorectal tumor samples and their adjacent tissues. In this project, we organized a two-stage approach including bioinformatics analysis for organization of a potential hypothetical pathway and then performing laboratory experiments.

The present study provided compelling evidence that circ0009910 is a novel dysregulated circular RNA in CRC. Circ0009910 was significantly upregulated in colorectal tumor tissues compared with adjacent tissues. It is worth mentioning that the selection criterion of the expression level changes > 3× in our study does not necessarily result in identification of genes with critical activity in the pathogenesis of CRC or critical importance over the process of carcinogenesis.

Also, expressions of miR-145-5p and PEAK1 in colorectal tumors compared with adjacent tissues were down- and upregulated, respectively. On the other hand, the significant correlation between circ0009910, miR-145-5p, and PEAK1 was observed. ROC curve analysis illustrated that circ0009910 can be as a putative biomarker in CRC. GEO data and bioinformatics analysis along with experimental analysis reinforced the hypothesis that circ0009910 regulates PEAK1 by sponging of miR-145-5p.

## Conclusion

Altogether, our study identified this axis as a potential contributor in the pathogenesis of CRC. Further experiments would help in identification of its role as a therapeutic target in CRC.

## Supplementary Information

**Additional file 1: Supplementary Table 1.**

**Additional file 2: Supplementary Table 2.**

## Data Availability

The datasets supporting the conclusions of this article are available in https://www.ncbi.nlm.nih.gov/geo/. The data supporting the conclusions of this article are also included within supplementary tables 1 and 2.
